# Identification and characterization of *VC1123*, a novel gene required for colonization in *Vibrio cholerae*

**DOI:** 10.3389/fmicb.2026.1758776

**Published:** 2026-02-24

**Authors:** Xiuping Fu, Xinyu Wen, Yuanyuan Yang, Weili Liang, Bo Pang, Xu Li, Baowei Diao, Jie Li, Jingyun Zhang, Biao Kan

**Affiliations:** 1 State Key Laboratory of Infectious Disease Prevention and Control, National Institute for Communicable Disease Control and Prevention, Chinese Center for Disease Control and Prevention, Beijing, China; 2 School of Light Industry Science and Engineering, Beijing Technology and Business University, Beijing, China

**Keywords:** intestinal colonization, neonatal mouse model, OmpT, OmpU, *Vibrio cholerae*, virulence regulation

## Abstract

**Introduction:**

A novel lineage of serogroup O1 El Tor *Vibrio cholerae*, genetically distinct from the seventh-pandemic strain, has recently emerged in China and has been associated with diarrheal outbreaks. However, the molecular basis underlying its virulence potential remains incompletely understood. This study aimed to identify virulence-associated determinants in the new lineage through comparative transcriptomic and functional analyses.

**Methods:**

We compared the transcriptomes of two new-lineage isolates—VC6050 (*ctxAB^+^*) and VC6055 (*ctxAB^−^*)—with the representative seventh-pandemic strain N16961 (*ctxAB^+^*) following growth in virulence-inducing (AKI) and non-inducing (LB) media. Differential expression patterns were analyzed, with particular focus on the toxigenic new-lineage strain VC6050 and N16961 under AKI conditions. A highly expressed gene shared across all three strains, *VC1123*, was selected for functional characterization. A targeted deletion mutant (Δ*VC1123*) was constructed in N16961, and its intestinal colonization capacity was evaluated in the neonatal mouse model. Complementation analysis was performed to confirm phenotype specificity. To investigate the underlying regulatory mechanisms, comparative RNA-seq was conducted between wild-type and Δ*VC1123* strains grown in LB medium.

**Results:**

Under AKI conditions, VC6050 exhibited more pronounced repression of chemotaxis- and biofilm-associated genes compared with N16961. Among genes highly expressed across all strains in AKI, *VC1123* was selected for further study. Deletion of *VC1123* significantly enhanced intestinal colonization in neonatal mice, and this phenotype was fully restored upon genetic complementation. Transcriptomic analysis revealed that *VC1123* deletion led to marked upregulation of the outer membrane porin gene ompU and concomitant downregulation of *ompT*, without affecting expression of the master regulators *toxR* or toxS. Given that *ompU* enhances bile resistance, adhesion, and intestinal survival, whereas *ompT* impairs gut colonization, these transcriptional alterations provide a mechanistic explanation for the enhanced colonization phenotype.

**Discussion:**

Our findings demonstrate that *VC1123* functions as a negative regulator of intestinal colonization in *V. cholerae*. The data suggest that *VC1123* modulates *ompU* and *ompT* expression through a pathway independent of the canonical ToxR/ToxS regulatory system. This study uncovers a previously underexplored layer of virulence regulation mediated by a conserved gene of unknown function and provides new insight into adaptive strategies employed by emerging *V. cholerae* lineages.

## Introduction

Cholera is a severe intestinal infectious disease caused by *Vibrio cholerae*. According to the World Health Organization, over 560,000 cases and more than 6,000 deaths were reported globally across 60 countries in 2024 (World Health Organization, 2025). *V. cholerae* is a Gram-negative bacterium that naturally resides in aquatic environments and associates with plankton. Infection occurs via the oral route, followed by colonization of the small intestinal epithelium. Intestinal colonization depends on the toxin-co-regulated pilus (TCP), which also serves as the receptor for the CTXφ bacteriophage (Gutierrez-Rodarte et al., 2019).

*V. cholerae* produces cholera toxin (CT), an ADP-ribosylating toxin that elevates intracellular cyclic AMP (cAMP) levels in host enterocytes, leading to massive secretory diarrhea and dehydration (Lebens and Holmgren, 1994). The genes encoding CT (*ctxA* and *ctxB*) are located on the lysogenic CTXφ bacteriophage. The core region of CTXφ includes *ctxA*, *ctxB*, *zot*, *cep*, *ace*, and *orfU*, and can be horizontally transferred between toxigenic and non-toxigenic strains (Waldor and Mekalanos, 1996).

Expression of the major virulence factors TCP and CT is coordinately regulated by the ToxR/ToxS transcriptional activator system (Miller et al., 1989; Herrington et al., 1988). ToxR, the first identified virulence gene regulator in *V. cholerae*, forms a complex with TcpP to activate *toxT*, which encodes a cytoplasmic transcriptional activator that directly induces expression of *ctxAB*, *tcp*, and accessory colonization factor genes (Miller and Mekalanos, 1985; Kovacikova and Skorupski, 1999; Haas et al., 2015; Richard et al., 2010). Independently of TcpP and ToxT, ToxR also modulates the expression of two outer membrane porins: it activates *ompU* transcription and represses *ompT* (Chakrabarti et al., 1996; Champion et al., 1997; Li et al., 2000; Crawford et al., 1998). Both OmpU and OmpT have been implicated in intestinal colonization (Provenzano and Klose, 2000).

This ToxR-dependent virulence cascade is robustly induced in the host intestinal environment, whereas *in vitro* induction requires specific, artificial culture conditions (Ross, 1996). To mimic key physicochemical cues of the small intestine—such as low oxygen tension, bicarbonate, pH, and osmolarity—the AKI culture system was established in 1986 (Iwanaga et al., 1986). Under AKI conditions, the *V. cholerae* virulence regulatory network is effectively activated, making this system a widely used model for studying virulence gene expression and regulation.

Although more than 200 *V. cholerae* serogroups have been identified, only toxigenic strains of serogroups O1 and O139 have caused epidemic cholera (Albert et al., 1993; Devault et al., 2014). Recently, a novel lineage of O1 El Tor *V. cholerae*, genetically distinct from the seventh pandemic strain, was identified in China and associated with diarrheal outbreaks (Yan et al., 2022). Our prior work showed that this new lineage induces significantly lower levels of inflammatory cytokines and chemokines compared to the classical pandemic strain (Fu et al., 2025). The new lineage contains toxigenic strains but is genetically distinct from the seventh pandemic strain. It remains unclear whether its virulence expression resembles that of the pandemic strains.

In this study, the new lineage strains VC6050 (*ctxAB*^+^) and VC6055 (*ctxAB*^−^), as well as the representative seventh pandemic strain N16961 (*ctx*AB^+^), were selected and cultured in AKI medium or LB medium. Then, their transcriptomes were compared to identify similarities and differences in virulence—factor expression between the new lineage and the pandemic strains, and to determine potential virulence factors. In addition, we screened candidate genes for functional validation to pinpoint potential virulence factors involved in *V. cholerae* pathogenesis.

## Materials and methods

### Bacterial strains

The following strains were used in this study: the seventh-pandemic strain N16961 (serogroup O1, El Tor biotype, *ctxAB*^+^); its isogenic deletion mutants N16961-Δ*VC1123*, the complemented strain N16961-Δ*VC1123*/c*VC1123*; and two isolates from the newly identified lineage, VC6050 (serogroup O1, *ctxAB*^+^) and VC6055 (serogroup O1, *ctxAB*^−^).

### Media and culture conditions

For *in vitro* growth experiments, strains N16961, VC6050, and VC6055 were cultured in either AKI medium (1.5% Bacto peptone, 0.4% yeast extract, 0.5% NaCl, 0.4% NaHCO₃, pH 7.4) or standard LB broth (1% tryptone, 0.5% yeast extract). Cultures were prepared in biological triplicate for each condition. In the AKI induction protocol, bacteria were first grown statically at 37 °C for 4 h to reach an OD_600_ of ~1.0, followed by continued incubation under shaking conditions (200 rpm, 37 °C) for an additional 6 h to induce virulence gene expression.

### Growth curve analysis

To assess whether deletion of *VC1123* affects the growth kinetics of *V. cholerae*, strains N16961 (wild type), N16961-Δ*VC1123*, and the complemented strain N16961-Δ*VC1123*/c*VC1123* were pre-cultured in LB broth at 37 °C with shaking (200 rpm) for 5 h to reach an OD_600_ of ~1.0. These cultures were then diluted 1:100 into fresh LB broth and grown in triplicate under both aerobic and anaerobic conditions at 37 °C shaking at 200 rpm. OD_600_ was measured hourly using a spectrophotometer. Growth curves represent the mean ± standard deviation of three biological replicates.

### Total RNA extraction and RNA sequencing

*V. cholerae* strains N16961, VC6050, and VC6055 were cultured in either AKI medium or standard LB broth until they reached an OD_600_ of approximately 1.0. *V. cholerae* strains N16961 and N16961-Δ*VC1123* were cultured in LB medium until they reached an OD_600_ of approximately 1.0.

Then, total RNA was extracted from bacterial cultures grown in AKI and LB media using TRIzol^®^ Reagent (Thermo Fisher Scientific), and genomic DNA was removed according to our previous experimental procedures (Fu et al., 2014).

All experiments were conducted with three independent biological replicates per sample. An rRNA Depletion Kit for Mixed Bacterial Samples (lexogen, United States) was used. Then, all mRNAs were first broken into short (200 nt) fragments by adding fragmentation buffer. Secondly, double-stranded cDNA was synthesized with random hexamer primers (Illumina). Then, the synthesized cDNA was subjected to end—repair, phosphorylation, and ‘A’ base addition according to Illumina’s library construction protocol. An RNA—seq transcriptome library was prepared following Illumina^®^ Stranded mRNA Prep, Ligation (San Diego, CA) using total RNA. A paired—end RNA—seq library was sequenced with the Illumina Novaseq Xplus (Illumina Inc., San Diego, CA, United States).

The original images were processed into sequences, and base—calling and quality value calculations were performed. Clean reads were obtained by removing low-quality sequences, reads with more than 10% of N bases (unknown bases), and reads containing adaptor sequences.

### Differential expression analysis and functional enrichment

Transcript abundance was quantified as transcripts per million (TPM), and gene counts were estimated using RSEM. Differentially expressed genes (DEGs) between conditions were identified using DESeq2 implemented in R (v4.3.0) (Love et al., 2014).

Functional enrichment analysis was performed to identify Kyoto Encyclopedia of Genes and Genomes (KEGG) pathways among the DEGs. KOBAS 2.0^1^ is used to identify statistically significantly enriched pathway using Fisher’s exact test. Enrichment significance was assessed against the whole-transcriptome background using FDR-corrected *p*-values, with a threshold of *p* ≤ 0.05.

### Reverse transcription quantitative real-time PCR (RT-qPCR)

To validate RNA-seq results, six DEGs were selected for RT-qPCR analysis. Quantitative PCR was performed according to our previous experimental procedures (Fu et al., 2025). Gene expression levels were normalized to the housekeeping gene *gap* (encoding glyceraldehyde-3-phosphate dehydrogenase) and calculated using the 2^−ΔΔCq^ method. Each qPCR reaction was run in technical triplicate, and the entire experiment was independently repeated twice. Primer sequences are listed in Supplementary Table 1.

To further verify whether the expression of ompU and ompT also differed between the wild-type strain and the *VC1123* mutant under AKI conditions, rather than only in LB medium, *V. cholerae* strains N16961 and N16961-Δ*VC1123* were cultured in AKI medium for 4 h to reach an OD_600_ of ~1.0. Total RNA was then extracted, and RT-qPCR analysis was performed. Primers for *ompU* are listed in Supplementary Table S1, while primers for ompT were as follows: Forward primer: 5′-CGCCAGTGTTCGCTTCTTG-3′ and Reverse primer: 5′-AGGTCTTCGTTACGGCACACTG-3′.

PCR amplification was carried out with an initial denaturation at 95 °C for 30 s, followed by 40 cycles of denaturation at 95 °C for 5 s and annealing at 60 °C (or the corresponding primer Tm) for 30 s.

### Construction of *VC1123* deletion and complementation strains

The nucleotide sequence of the *V. cholerae VC1123* gene was retrieved from the NCBI RefSeq database. Homologous sequences were identified using BLASTN with an E-value threshold of 1 × 10^−5^.

A clean, in-frame deletion mutant of *VC1123* was constructed in the parental strain N16961 via allelic exchange using the suicide vector pWM91. Briefly, DNA fragments corresponding to the upstream (amplified with primers VC1123F1/VC1123R1) and downstream (amplified with primers VC1123F2/VC1123R2) regions of *VC1123* were PCR-amplified from N16961 genomic DNA. These fragments were assembled into XhoI/SacI-digested pWM91 using the ClonExpress^®^ Ultra One Step Cloning Kit V3 (Vazyme), yielding the allelic exchange plasmid pWM91-Δ*VC1123*. The plasmid pWM91-*ΔVC1123* was then transformed into the *Escherichia coli* strain SM10 *pir* and subsequently transferred to the strain N16961 by conjugation. The transconjugants were selected on LB agar plates containing streptomycin (100 μg/mL) and ampicillin (100 μg/mL). The clones were streaked on LB agar plates with 8% sucrose and without NaCl at 22 °C. After 48 h, double—crossover recombination mutants were selected and verified by sequencing to generate the mutant strain, N16961-Δ*VC1123*.

For genetic complementation, the full-length *VC1123* open reading frame (ORF) was amplified from N16961 DNA using primers H1123F and H1123R. The amplicon was cloned into the SpeI/NdeI-digested vector pSRKKtc using the same ClonExpress^®^ cloning kit, generating the plasmid pSRKKtc-VC1123. The construct was confirmed by restriction digestion and sequencing. The plasmid was then electroporated into N16961-Δ*VC1123*, producing the complemented strain N16961-Δ*VC1123*/pSRKKtc-VC1123 (abbreviated as N16961-Δ*VC1123*/c*VC1123*).

All primers used for mutant and complementation construct generation are listed in Supplementary Table S2.

### Infant mouse colonization competition assays

Strains N16961, N16961-Δ*VC1123*, and the complemented strain N16961-Δ*VC1123*/c*VC1123* were grown overnight on LB agar plates at 37 °C. Cells were harvested and resuspended in phosphate-buffered saline (PBS) to an OD_600_ of 1.0 (~1 × 10^9^ CFU/mL). For each competition assay, ~1 × 10^5^ CFU of either N16961-Δ*VC1123* or N16961-Δ*VC1123*/c*VC1123* was mixed with an equal number of wild-type N16961 cells in a total volume of 50 μL (1:1 input ratio). The actual the input ratios (mutant to wild type) of the inoculum was verified by serial dilution and plating on LB agar.

Five- to six-day-old C57BL/6 neonatal mice, separated from their dams for 2–3 h prior to infection, were intragastrically inoculated with 50 μL of the bacterial mixture. At 24 h post-inoculation, pups were euthanized, and the entire small intestine (SI) from each mouse was aseptically removed and placed individually into 5 mL of cold PBS. Each intestinal sample was homogenized separately. Serial dilutions were made and plated on LB agar plates supplemented with streptomycin 100μg/ml for calculation of the output ratios (mutant to wild type). Genomic DNA was extracted directly from 10-fold diluted homogenates (see below) for digital PCR (dPCR)-based quantification. Each experimental group included five mice, and two independent biological replicates were performed. The competitive index (CI) was calculated for each mouse as:


$$\mathrm{CI}=\frac{{\left(\text{mutant}/\text{wild type}\right)}_{\text{output}}}{{\left(\text{mutant}/\text{wild type}\right)}_{\text{input}}}$$

A CI < 1 indicates impaired colonization fitness of the mutant relative to the wild type. All assays were repeated in at least two independent experiments.

### Digital PCR (dPCR) quantification of bacterial loads

Intestinal colonization levels were quantified using dPCR on a QIAcuity One system (Qiagen, Hilden, Germany). Primers (Supplementary Table S2) were designed to target two genomic loci: (i) a unique sequence within the deleted region of *VC1123* (absent in the Δ*VC1123* mutant but present in wild-type and complemented strains), and (ii) the single-copy housekeeping gene *recA*, which is conserved in all strains and serves as a proxy for total *V. cholerae* genome equivalents. Because both targets are present in one copy per chromosome, dPCR-derived copy numbers directly reflect bacterial genome counts. The *recA* signal represents the total bacterial population (wild type + mutant), whereas amplification of the *VC1123*-specific locus specifically quantifies wild-type (or complemented) cells. The mutant population was inferred by subtracting the wild-type count from the total (*recA*) count.

Each 40 μL dPCR reaction contained 13.3 μL of 3 × QIAcuity EG Master Mix, 0.4 μM of each primer, and 4 μL of template DNA. Reactions were loaded onto QIAcuity Nanoplate 26 k chips, sealed, and thermocycled under the following conditions: 95 °C for 2 min; 40 cycles of 95 °C for 15 s, 58 °C for 15 s, and 72 °C for 15 s; followed by a final hold at 40 °C for 5 min. Fluorescence data were acquired and analyzed using QIAcuity Software Suite v2.0 to determine target copies per microliter.

Template DNA was prepared from intestinal homogenates as follows: small intestines were homogenized in 5 mL PBS, diluted 1:10 in PBS, boiled for 10 min, and centrifuged at 12,000 × *g* for 2 min; the supernatant was used directly as PCR template. Meanwhile, to evaluate the potential influence of DNA from nonviable bacteria on the digital PCR (dPCR) assay, single colonies recovered from plates following serial dilution were analyzed by dPCR to identify their genotypes (wild-type, deletion mutant, or complemented strain). The results demonstrated that the competitive index (CI) determined from single-colony analysis were not significantly different from intestinal homogenates obtained by dPCR. Accordingly, the subsequent presentation and discussion of results are based directly on the dPCR data. All dPCR reactions were performed in technical triplicate. Primer sequences are listed in Supplementary Table S3.

### Bile salt tolerance assay

To assess whether deletion of *VC1123* increased bile salt tolerance, *V. cholerae* N16961 and N16961-Δ*VC1123* were pre-cultured in LB broth at 37 °C with shaking (200 rpm) for 5 h to reach an OD_600_ of ~1.0. The cultures were subsequently diluted 1:100 into fresh LB medium supplemented with 0.3% or 0.5% bile salts, with LB medium without bile salts used as the control. Cultures were incubated at 37 °C with shaking at 200 rpm for 12 h. OD_600_ was measured hourly using a spectrophotometer. Growth curves represent the mean ± standard deviation of three biological replicates. All experiments were performed with three independent biological replicates per sample.

## Results

### Transcriptomic profiling of *Vibrio cholerae* strains under virulence-inducing (AKI) and non-inducing (LB) conditions

To investigate the transcriptional responses of pandemic and emerging *V. cholerae* lineages to virulence-inducing conditions, we performed RNA sequencing on the seventh-pandemic strain N16961 and two isolates from the newly identified lineage —VC6050 (*ctxAB*^+^) and VC6055 (*ctxAB*^−^)—cultured in AKI medium (virulence-inducing) and standard LB broth (control). A total of 18 libraries (3 strains × 2 conditions × 3 biological replicates) yielded 67.97 Gb of high-quality sequencing data, with each sample generating >2.94 Gb of clean data and a Q30 base percentage exceeding 96.56%.

### Global transcriptomic responses to AKI induction

**Figure 1:**
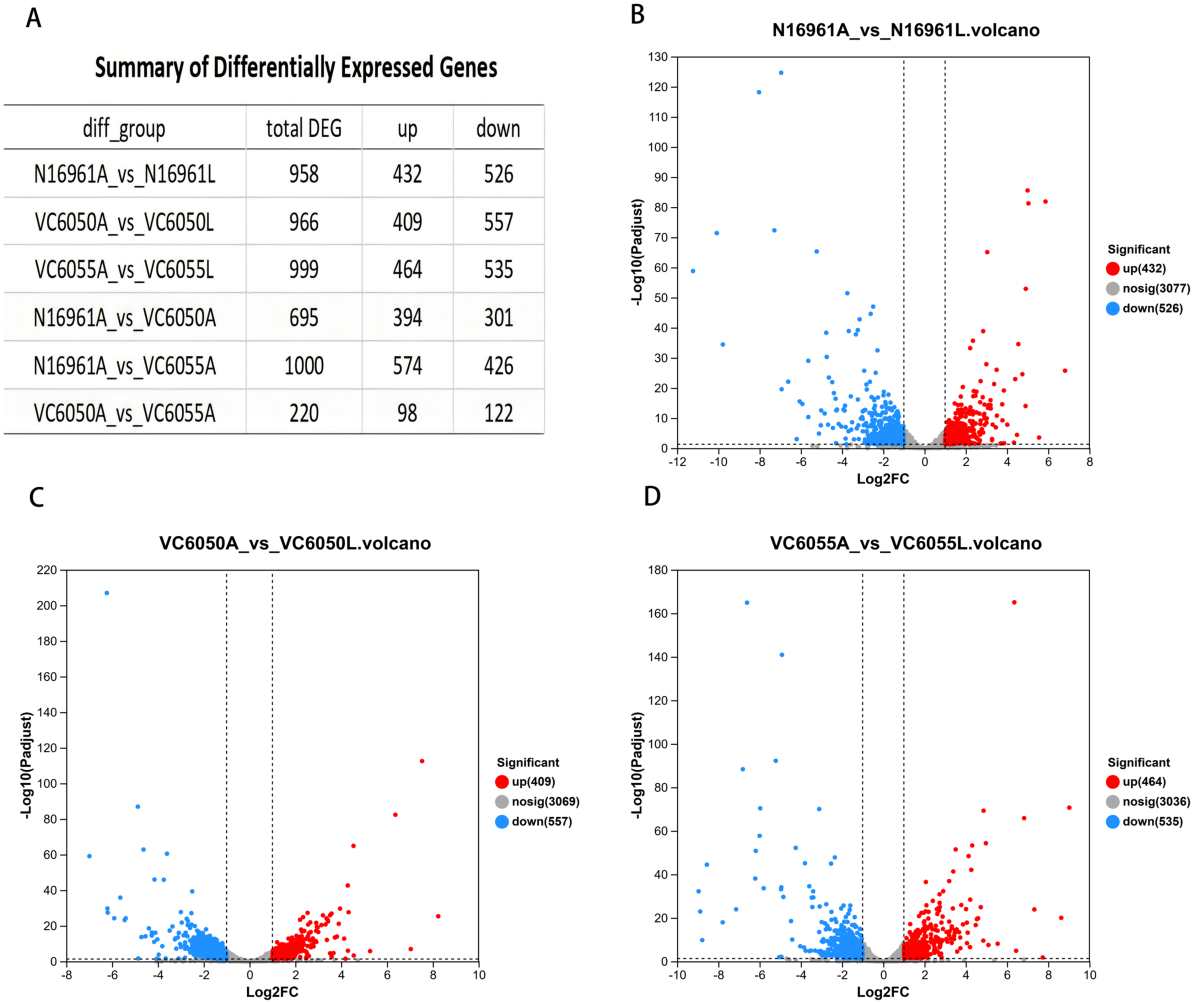
Results of transcriptome analysis. **(A)** Summary of differentially expressed genes. **(B–D)** Volcano plot of differentially expressed genes (DEGs) for groups N16961, VC6050, and VC6055 in AKI compared to the control group (LB), respectively.

Relative to LB-grown controls, AKI culture induced extensive changes in gene expression in all three strains: 958, 966, and 999 DEGs were identified in N16961, VC6050, and VC6055, respectively (Figure 1).

**Figure 2:**
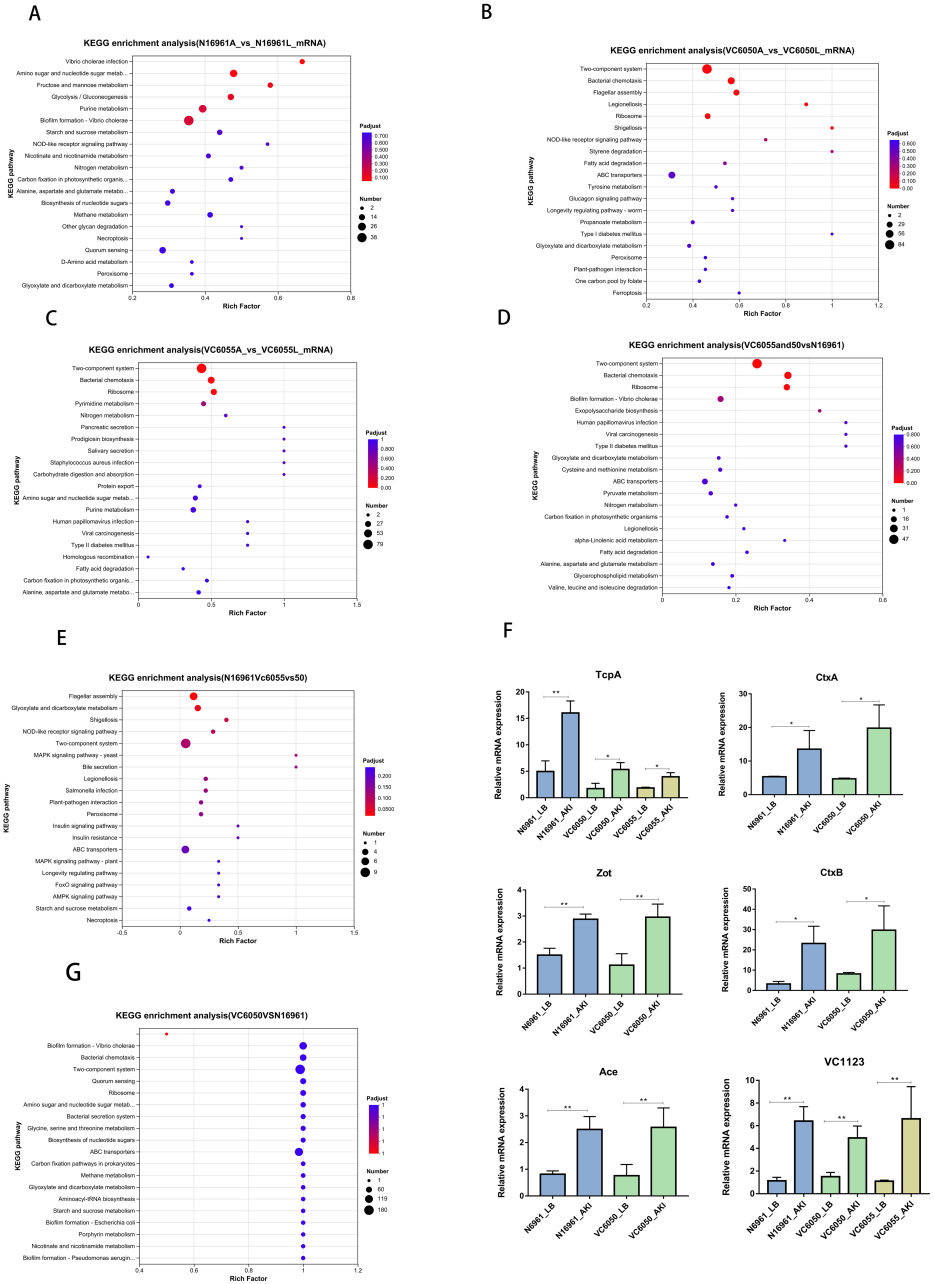
KEGG enrichment analysis results. **(A–C)** The top 20 pathways ranked by KEGG enrichment analysis for groups N16961, VC6050, and VC6055 compared to the control group (LB), respectively. **(D)** The top 20 pathways ranked by KEGG enrichment analysis for N16961 versus VC6050/VC6055. **(E)** The top 20 pathways ranked by KEGG enrichment analysis for N16961/VC6050 versus VC6055. **(F)** The expression of genes, with particular attention given to (*tcpA, ctxAB, zot, ace,* and *VC1123*) [*n* = 3, mean ± standard deviation (SD)]. * *p* < 0.01, ** *p* < 0.001; unpaired two-tailed *t*-test. **(G)** The top 20 pathways ranked by KEGG enrichment analysis for VC6050 versus N16961.

In N16961, DEGs were significantly enriched in pathways including *Vibrio cholerae* infection, amino sugar and nucleotide sugar metabolism, fructose and mannose metabolism, glycolysis/gluconeogenesis, and biofilm formation (Figure 2A). In strain VC6050, DEGs were mainly enriched in the two-component system, bacterial chemotaxis, ribosome, flagellar assembly, and ABC transporters (Figure 2B). For strain VC6055, DEG enrichment was observed predominantly in the two-component system, bacterial chemotaxis, ribosome, pyrimidine metabolism, and nitrogen metabolism (Figure 2C).

Notably, bacterial chemotaxis and biofilm formation pathways were consistently downregulated across all three strains under AKI conditions. Key chemotaxis genes—including *cheW*, *cheR*, *cheB*, *cheD*, *mcpH*, and *cher2*—showed pronounced transcriptional repression (Figure 2F). Conversely, canonical virulence determinants were upregulated: *tcpA* was induced in all strains, while *ctxAB* (cholera toxin), *zot* (zonula occludens toxin), and *ace* (accessory cholera enterotoxin) were specifically upregulated in the toxigenic strains N16961 and VC6050 (Figure 2F).

### Comparative analysis among strains under AKI conditions

Pairwise comparisons of the three strains in AKI medium revealed extensive transcriptional divergence: N16961 vs. VC6050, 695 DEGs; N16961 vs. VC6055, 1,000 DEGs; VC6050 vs. VC6055, 220 DEGs (Figure 1). Relative to the N16961 AKI group, the AKI-cultured new-lineage strains VC6050 and VC6055 shared 342 DEGs, which were significantly enriched in the two-component system, bacterial chemotaxis, and biofilm formation (Figure 2D). When comparing only the toxigenic strains (N16961 and VC6050) against the non-toxigenic VC6055, 84 DEGs were shared and enriched in flagellar assembly and bile secretion pathways (Figure 2E).

Focusing specifically on the comparison between VC6050 and N16961 under AKI conditions, we identified 301 upregulated and 394 downregulated genes in VC6050. These DEGs were predominantly associated with bacterial chemotaxis, biofilm formation, and two-component signaling. Importantly, the magnitude of downregulation in chemotaxis- and biofilm-related genes was greater in VC6050 than in N16961 (Figure 2G).

### Deletion of *VC1123* enhances intestinal colonization by *Vibrio cholerae* in infant mice

**Figure 3:**
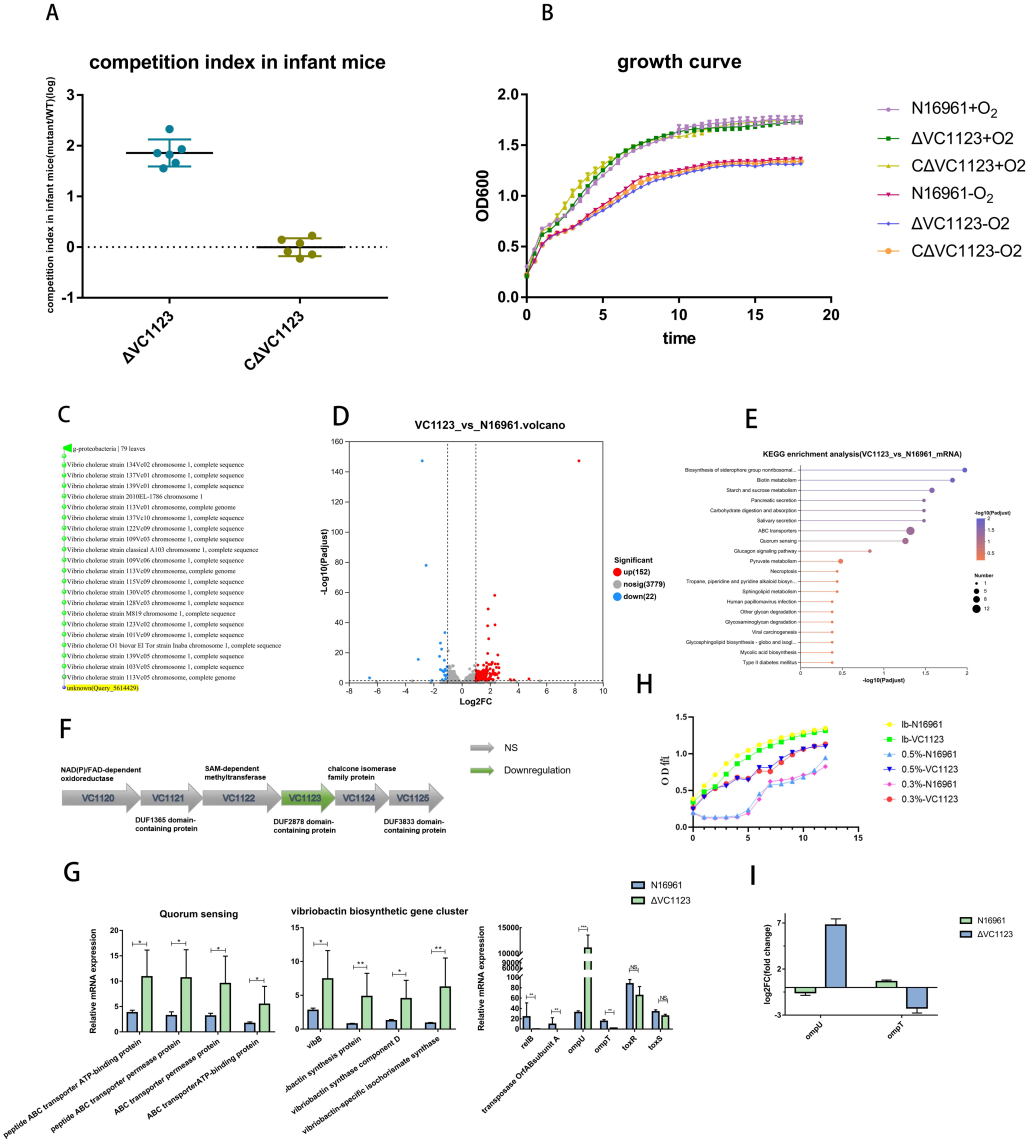
Deletion of the *VC1123* gene enhances the colonization ability of *V. cholerae* in the infant mouse intestine. **(A)** Colonization competition assays in infant mice. **(B)** Growth curve analysis of the *V. cholerae* strain N16961, N16961-Δ*VC1123,* and N16961-Δ*VC1123/cVC1123* in aerobic or anaerobic conditions. **(C)** Homologous sequences were identified using BLASTN of the *V. cholerae VC1123* gene with an *E*-value threshold of 1e-5. **(D)** Volcano plot of differentially expressed genes (DEGs) for N16961-Δ*VC1123* versus N16961. **(E)** The top 20 pathways ranked by KEGG enrichment analysis for N16961-Δ*VC1123* versus N16961. **(F)** The transcription of *VC1123* neighboring genes (*VC1120–VC1125*). **(G)** The expression of *ompU*, *ompT, toxR, toxS and* several genes related to iron uptake and the quorum sensing system [*n* = 3, mean ± standard deviation (SD)] * *p* < 0.01, ** *p* < 0.001, *** *p* < 0.0001, **** *p* < 0.00001; unpaired two-tailed *t*-test. **(H)** Bile salt tolerance assay. *V. cholerae* N16961 and N16961-Δ*VC1123* were incubated in LB medium supplemented with 0.3% or 0.5% bile salts, with LB medium without bile salts used as the control. **(I)** The relative transcript levels of *ompU* and *ompT* in groups of N16961 and the VC1123 mutant under AKI conditions as determined by RT-qPCR.

The gene VC1123, which was highly expressed across all three strains—N16961, VC6050, and VC6055 (Figure 2F)—was selected for functional characterization based on several considerations beyond transcriptional abundance alone. VC1123 is a 540-bp open reading frame in *V. cholerae* that encodes a protein containing a domain of unknown function (DUF), making it an attractive candidate for uncovering previously unrecognized regulatory mechanisms. In addition, VC1123 is highly conserved among *V. cholerae* isolates, as evidenced by multiple-sequence alignment showing highly conserved amino acid sequences in O1 and O139 serogroups as well as diverse non-O1/non-O139 environmental strains (Figure 3C), suggesting an important biological role. Moreover, its consistently high expression in both the new-lineage isolates and the seventh-pandemic reference strain under virulence-inducing conditions indicates that VC1123 may participate in core processes related to host adaptation rather than strain-specific traits. Collectively, these features distinguished VC1123 from other AKI-induced genes and provided the rationale for selecting it for further functional investigation.

Efforts to construct *VC1123* deletion mutants in the new-lineage strains VC6050 and VC6055 were unsuccessful, as no transconjugants could be recovered on dual-antibiotic selection plates following conjugation. During the deletion of other genes in the new-lineage strains, the suicide plasmid similarly failed to be transferred into the recipient cells; the same phenomenon was also observed in our previous gene deletion experiments using environmental isolates. Consequently, only the *VC1123* deletion mutant in the N16961 background (N16961-Δ*VC1123*) was obtained. A complemented strain, N16961-Δ*VC1123*/c*VC1123*, was also generated.

To assess the role of *VC1123* in intestinal colonization, competitive infection assays were performed in 5–6-day-old C57BL/6 neonatal mice. Equal mixtures (1:1 ratio) of wild-type N16961 with either N16961-Δ*VC1123* or N16961-Δ*VC1123*/c*VC1123* were administered intragastrically. Consistently, 24 h post-inoculation, the Δ*VC1123* mutant exhibited an 86.34-fold increase in intestinal colonization relative to the wild-type strain (Figure 3A). In contrast, the complemented strain showed colonization levels comparable to wild-type, with a mean CI of 1.06 (Figure 3A), indicating that *VC1123* negatively regulates intestinal colonization.

To rule out growth defects as a confounding factor, we compared the *in vitro* growth kinetics of the mutant and wild-type strains under both aerobic and anaerobic conditions. No significant differences in growth rate were observed (Figure 3B), indicating that the enhanced colonization was not due to altered bacterial fitness in broth culture.

To explore the molecular basis of this phenotype, we performed RNA sequencing on N16961 and N16961-Δ*VC1123* cultured in LB medium to mid-log phase (5 h). Deletion of *VC1123* did not affect transcription of neighboring genes (*VC1120–VC1125*; Figure 3F), suggesting minimal polar effects on the surrounding genes. Differential expression analysis identified 174 DEGs in the mutant relative to wild-type, of which 152 were upregulated and 22 were downregulated (Figure 3D). KEGG pathway enrichment revealed that these DEGs were primarily associated with quorum sensing, biosynthesis of siderophore group nonribosomal peptides, and biotin metabolism (Figure 3E).

Notably, the outer membrane protein gene *ompU* was dramatically upregulated (315.6-fold) in the Δ*VC1123* strain, while *ompT* was downregulated (8.47-fold). In contrast, the virulence-associated regulators *toxR* and *toxS* showed no significant changes in expression. Additionally, *relB* and the transposase *orfAB* subunit A were strongly downregulated (90.9-fold and 66.67-fold, respectively).

Genes involved in the quorum sensing, including ABC transporter permease protein, ABC transporter ATP-binding protein, peptide ABC transporter permease protein, and peptide ABC transporter ATP-binding protein, were significantly upregulated, with an average increase of 2.74-fold. Furthermore, multiple genes critical for iron acquisition via vibriobactin biosynthesis were significantly induced, including vibriobactin-specific 2,3-dihydroxybenzoate-AMP ligase, vibriobactin synthesis protein, vibriobactin synthase component D, and vibriobactin-specific isochorismate synthase (Figure 3G).

To evaluate whether deletion of VC1123 increased bile salt tolerance, *V. cholerae* N16961 and N16961-ΔVC1123 were cultured in LB medium supplemented with different concentrations of bile salts, and bacterial growth was compared under the indicated conditions. The results showed that, compared with the N16961 strain, N16961-ΔVC1123 exhibited a pronounced growth advantage at bile salt concentrations of 0.3 and 0.5% (Figure 3H).

To further verify whether the expression of *ompU* and *ompT* also differed between the wild-type strain and the *VC1123* mutant under AKI conditions, rather than only in LB medium, RT-qPCR analysis was performed. The results showed that, under AKI culture conditions, *ompU* was significantly upregulated, whereas *ompT* was significantly downregulated in the *VC1123* deletion mutant compared with the wild-type strain (Figure 3I), consistent with the observations in LB medium.

**Figure 4:**
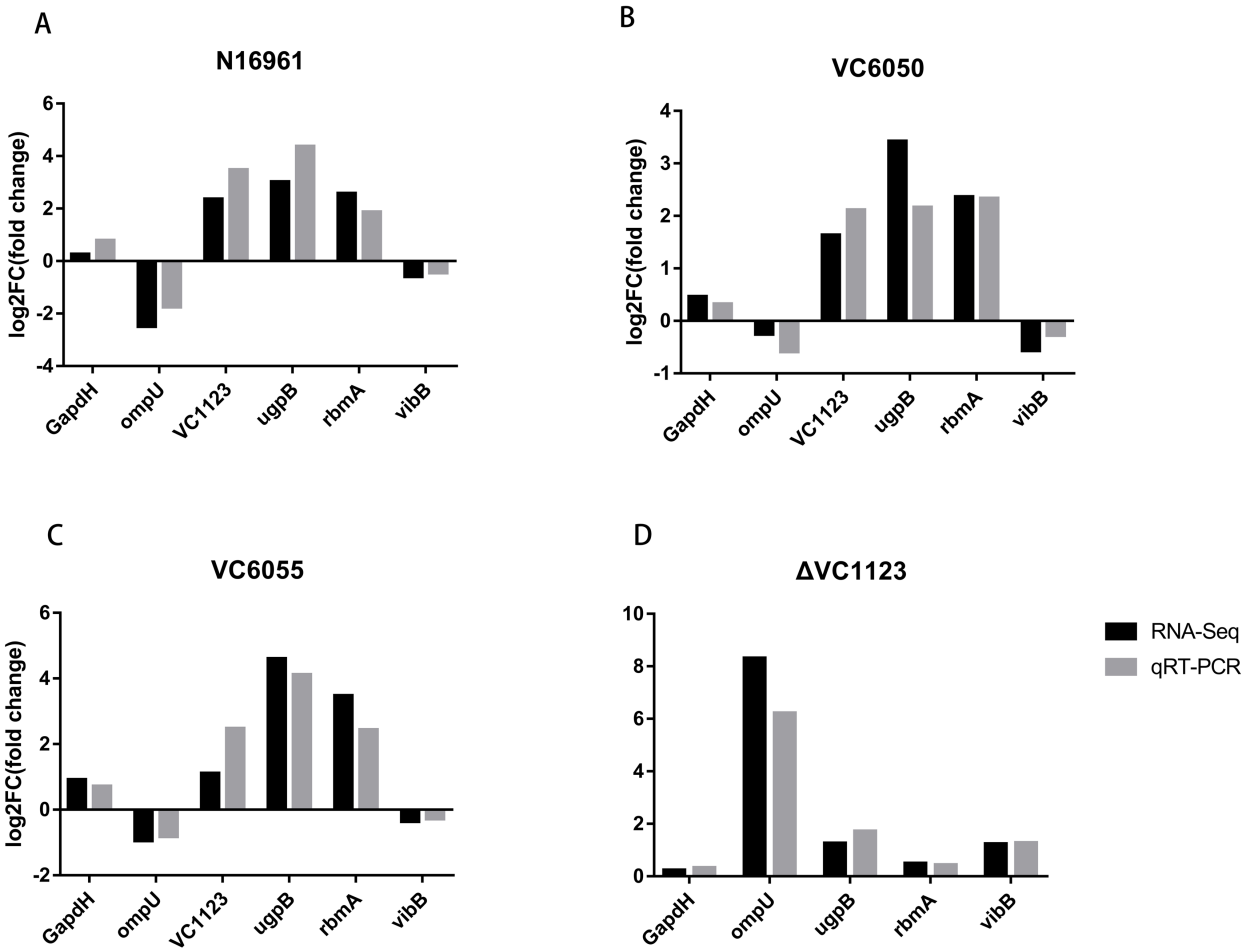
RT-qPCR validation of transcriptome data. **(A–C)** The relative transcript levels of six DEGs were consistent between RNA-seq and RT-qPCR in groups of N16961, VC6050, and VC6055 in AKI medium. **(D)** The relative transcript levels of five DEGs were consistent between RNA-seq and RT-qPCR in Δ*VC1123*. Black bars represent RNA-seq results, while gray bars represent RT-qPCR results.

To validate the RNA-seq findings, six DEGs were selected for RT-qPCR analysis. The expression trends observed by RT-qPCR were fully consistent with those from transcriptomic data (Figure 4), confirming the reliability of the sequencing results.

## Discussion

In this study, we employed RNA-seq to characterize the transcriptional responses of pandemic and emerging *V. cholerae* lineages under virulence-inducing (AKI) versus non-inducing (LB) conditions. Consistent with the established role of AKI medium in mimicking the host intestinal environment, all three strains—N16961 (*ctxAB^+^*), VC6050 (*ctxAB^+^*), and VC6055 (*ctxAB^−^*)—exhibited strong upregulation of the major colonization factor *tcpA*. Moreover, the canonical virulence genes *ctxAB*, *zot*, and *ace* were specifically induced in the two toxigenic strains (N16961 and VC6050), confirming that AKI conditions effectively activate the core virulence regulon.

Notably, genes associated with bacterial chemotaxis and biofilm formation were consistently downregulated across all three strains in AKI medium. This transcriptional shift suggests a global reallocation of cellular resources away from environmental persistence mechanisms toward host colonization programs—a strategy previously linked to reduced intracellular cyclic diguanylate (c-di-GMP) levels (Matson et al., 2007). In *V. cholerae*, the phosphodiesterase VieA lowers c-di-GMP, thereby repressing *vps*-mediated biofilm formation and promoting expression of colonization factors such as TCP (Tischler and Camilli, 2004). Our data align with this model, reinforcing the notion that low c-di-GMP states favor virulence over environmental adaptation.

Intriguingly, the toxigenic new-lineage strain VC6050 displayed more pronounced repression of chemotaxis- and biofilm-related genes than the classical pandemic strain N16961. We hypothesize that this enhanced downregulation may reflect an evolved strategy to minimize host immune detection or phage predation. For instance, reduced flagellar synthesis and biofilm production could lower immunogenicity and decrease susceptibility to phage recognition. Supporting this idea, our prior work demonstrated that these new-lineage isolates exhibit reduced sensitivity to the VP3 bacteriophage (Yuanxi et al., 2025), suggesting potential co-evolutionary adaptations favoring stealth during infection.

Building on the transcriptomic profiling, we identified *VC1123*—a gene highly expressed in all three strains—as a candidate modulator of virulence. *VC1123* encodes a conserved protein containing a DUF2878 (PF11086) domain and is annotated within the PhrR regulon (RegPrecise), implying regulation by the MerR-family transcription factor PhrR. Using N16961 as the parental strain, we generated an in-frame deletion mutant (N16961-Δ*VC1123*) and a complemented derivative. Competitive colonization assays in neonatal mice revealed that loss of *VC1123* conferred an enhancement in intestinal colonization, without altering in vitro growth under aerobic or anaerobic conditions. Furthermore, transcription of flanking genes (*VC1120–VC1125*) remained unchanged, ruling out polar effects. These findings collectively indicate that *VC1123* functions as a negative regulator of colonization.

RNA-seq analysis of the Δ*VC1123* mutant uncovered significant dysregulation of outer membrane porin genes: *ompU* was dramatically upregulated (315.6-fold), while *ompT* was downregulated (8.47-fold), despite no detectable changes in *toxR* or *toxS* expression. This observation is particularly significant given the well-established roles of OmpU and OmpT in *V. cholerae* pathogenesis (Valeru et al., 2012). OmpU enhances resistance to bile salts and cationic antimicrobial peptides, promotes adhesion to intestinal epithelial cells, and facilitates colonization in murine models (Provenzano and Klose, 2000; Duret and Delcour, 2006; Wibbenmeyer et al., 2002; Mathur and Waldor, 2004; Sperandio et al., 1995). In contrast, OmpT compromises bile tolerance and reduces fitness in the gut (Wibbenmeyer et al., 2002). Notably, anti-OmpU antibodies inhibit bacterial attachment in vitro and diminish colonization *in vivo* (Sperandio et al., 1995), and OmpU can also trigger IL-8 secretion from intestinal epithelial cells, potentially shaping host inflammatory responses (Yang et al., 2018). Moreover, OmpU has been implicated as a receptor for epithelial cell endocytosis and as a scaffold for cholera toxin packaging into outer membrane vesicles (OMVs), thereby stabilizing toxin activity in the intestine (Zingl et al., 2021).

Given that *toxR/toxS* expression was unaffected, our data suggest that *VC1123* modulates *ompU/ompT* through a ToxR-independent pathway. Potential mechanisms include direct or indirect regulation via alternative transcription factors, post-transcriptional control, or modulation by host- or microbiota-derived signals. For example, gut microbial metabolites have been shown to influence *ompU* expression and colonization efficiency (Qin et al., 2020). Additionally, the observed downregulation of *relB*—a component of the RelE/RelB toxin-antitoxin system—may contribute to the phenotype, as TA systems can impact biofilm formation and stress adaptation in other bacteria (Lemos et al., 2005), though its role in *V. cholerae* remains unclear. We hypothesize that *VC1123* may function as part of a PhrR-associated regulatory module that links environmental or redox sensing to outer membrane remodeling. Loss of *VC1123* disrupts this regulatory balance, leading to reciprocal regulation of OmpU and OmpT and thereby enhancing intestinal fitness independently of the canonical ToxR/ToxS pathway.

In summary, our findings reveal that *VC1123*, a highly conserved gene of unknown function, acts as a suppressor of intestinal colonization, likely by suppressing *ompU* and/or promoting *ompT* through a ToxR-independent mechanism. The enhanced virulence of the Δ*VC1123* mutant underscores the importance of fine-tuning outer membrane composition during host infection. Future studies should aim to (i) define the molecular function of the VC1123 protein, (ii) identify its regulatory targets and interacting partners, and (iii) determine whether *VC1123* expression is modulated by host or microbial cues in the gut. Such insights may uncover novel layers of virulence control in *V. cholerae* and inform strategies to disrupt colonization.

This study has several limitations. Functional analyses of *VC1123* were conducted exclusively in the N16961 genetic background, and thus it remains to be determined whether the observed effects are conserved across other seventh-pandemic or new-lineage *V. cholerae* strains. In addition, although deletion of *VC1123* resulted in reciprocal regulation of *ompU* and *ompT* and enhanced intestinal colonization, we have not directly demonstrated that porin imbalance is the causal driver of the colonization phenotype. Future studies using multiple strain backgrounds and targeted genetic epistasis analyses will be required to establish the generality of *VC1123* function and to definitively link OmpU/OmpT dysregulation to intestinal fitness.

## Data Availability

The datasets presented in this study can be found in online repositories. The names of the repository/repositories and accession number(s) can be found below: https://www.ncbi.nlm.nih.gov/genbank/, PRJNA1416472 and https://www.ncbi.nlm.nih.gov/genbank/, PRJNA1416559.

## Footnotes

1
http://kobas.cbi.pku.edu.cn
