# Supplementary material for: Identification and characterization of VC1123, a novel gene required for colonization in Vibrio cholerae
Source: Front Microbiol. 2026 Feb 24;17:1758776. doi: 10.3389/fmicb.2026.1758776 (PMC12971978; doi:10.3389/fmicb.2026.1758776)
Supplement: Supplementary file 1 [file Data_Sheet_1.pdf]

TABLE S1 primers used in the RT-qPCR reactions

Gene	qRT-PCR primer sequence (5‘ to 3’)	
	Forward	Reverse
gap	GAAGCAGGCGCGAAGAAAGTTG	TACGCGGAAAGCCATACCAG
ompU	CAGAGTACTTGGCAGCGTTTGTTT	GTGCTGAAGCTCGCCTATCTCTGA
VC1123	GCAATATGCTGCTTGGGTTAC	TGGCCTGCATACCAAGTAAA
ugpB	TACGACCTACCTGCCTTACT	AGCTGCGACACCTTTATCC
rbmA	TGGCAAGTAACGGTGGATAC	GCTTTGGCTGGGAAGTAGAT
vibB	ATCGCGGGCGGTACTGCTTATC	CACGCCATCCCCAATCACAAAA
