# Supplementary material for: Identification and characterization of VC1123, a novel gene required for colonization in Vibrio cholerae
Source: Front Microbiol. 2026 Feb 24;17:1758776. doi: 10.3389/fmicb.2026.1758776 (PMC12971978; doi:10.3389/fmicb.2026.1758776)
Supplement: Supplementary file 2 [file Data_Sheet_2.pdf]

**Table S2 Primers for mutant strain and complementary strain construction**

| Prime | Sequences |
|---|---|
| VC1123F1 | ggtaccgggccccccctcgagGCAGAAGCTTACATGGAAGGGT |
| VC1123R1 | gaagagtatgggaaGCCATGATTAATCTCGTTCAAAAG |
| VC1123F2 | catggcTTCCCATACTCTTCTGTGTCTCGC |
| VC1123R2 | agggaacaaaagctggagctcCCAGATCCGTTGATCCTTTTCC |
| H1123F | tcacacaggaaacagcatatgATGGCGTTAGTGGCGATATCA |
| H1123R | cagcccgggggatccactagtTTAGCGACGGCGGCGTAA |
