# Supplementary material for: Identification and characterization of VC1123, a novel gene required for colonization in Vibrio cholerae
Source: Front Microbiol. 2026 Feb 24;17:1758776. doi: 10.3389/fmicb.2026.1758776 (PMC12971978; doi:10.3389/fmicb.2026.1758776)
Supplement: Supplementary file 3 [file Data_Sheet_3.pdf]

TABLE S3 primers used in the dPCR reactions

Gene	Primer sequence（5'-3'）
vc1123	F: GCAATATGCTGCTTGGGTTAC
	R: TGGCCTGCATACCAAGTAAA
recA	F: GGTAACCCAGAAACTACCACTG
	R: CACCACTTCTTCGCCTTCTT
